# Unperturbed Posttranscriptional Regulatory Rev Protein Function and HIV-1 Replication in Astrocytes

**DOI:** 10.1371/journal.pone.0106910

**Published:** 2014-09-04

**Authors:** Ashok Chauhan

**Affiliations:** Department of Pathology, Microbiology and Immunology, and Department of Pharmacology, Physiology and Neuroscience, University of South Carolina School of Medicine, Columbia, South Carolina, United States of America; Institute of Molecular Genetics IMG-CNR, Italy

## Abstract

Astrocytes protect neurons, but also evoke proinflammatory responses to injury and viral infections, including HIV. There is a prevailing notion that HIV-1 Rev protein function in astrocytes is perturbed, leading to restricted viral replication. In earlier studies, our finding of restricted viral entry into astrocytes led us to investigate whether there are any intracellular restrictions, including crippled Rev function, in astrocytes. Despite barely detectable levels of DDX3 (Rev-supporting RNA helicase) and TRBP (anti-PKR) in primary astrocytes compared to astrocytic cells, Rev function was unperturbed in wild-type, but not DDX3-ablated astrocytes. As in permissive cells, after HIV-1 entry bypass in astrocytes, viral-encoded Tat and Rev proteins had robust regulatory activities, leading to efficient viral replication. Productive HIV-1 infection in astrocytes persisted for several weeks. Our findings on HIV-1 entry bypass in astrocytes demonstrated that the intracellular environment is conducive to viral replication and that Tat and Rev functions are unperturbed.

## Introduction

Astrocytes are neuroprotective cells in the brain that are important in HIV-1-mediated neuropathology, serving as inflammatory cells in response to viral products. Astrocytes also serve as HIV-1 reservoirs. In HIV-1-infected brain tissues, up to 19% of astrocytes carry viral DNA [1]–[3]. Restrictions at the HIV-1 entry level in astrocytes have been reported [4]–[8], and have suggested a compensatory viral entry mechanism [7] [8]. However, some studies have also suggested that there are intracellular restrictions on HIV-1 replication in astrocytes [9], with the presence of efficient early viral transcripts, but low levels of late transcripts being responsible for structural proteins [9]. Restrictions on HIV-1 replication in astrocytes have been attributed specifically to malfunction of the viral Rev protein [9]. Several cellular factors, among them Src-associated substrate in mitosis (Sam68), Tar RNA binding protein (TRBP), and protein kinase RNA-activated (PKR), have been implicated in unproductive HIV infection in astrocytes.

Two early HIV-1 regulatory proteins, Tat and Rev, which are produced upon multiple splicing from full-length viral transcripts, are indispensable for temporal regulation of the viral life cycle. Since unspliced and partially spliced viral transcripts are required in the cytoplasm for translation and packaging, HIV-1 must bypass the splicing and nuclear export of mRNA species. Nuclear export of these mRNA species is facilitated by HIV-1 protein Rev. This protein binds to the Rev-responsive element (RRE), which is present in all unspliced and partially spliced viral RNA transcripts [10]–[12]. More precisely, Rev interacts with a *cis*-acting viral RNA target sequence, a rev-responsive element (RRE), and chromosomal region maintenance (CRM1), a host cell protein that is a member of the karyopherin or importin/exportin family of nucleocytoplasmic transport factors [15]–[17]. CRM1 (exportin 1) specifically binds to a short leucine-rich motif in the Rev protein, which also functions as a nuclear export signal (NES). NES binding by CRM1 requires a cellular cofactor, Ran-GTP, and is enhanced by other cellular cofactors [156],[16]. This complex of factors is sensitive to leptomycin-B (LMB) which blocks Rev export by binding to CRM1 [18],[19].

Dead-box RNA helicases DDX1 and DDX3, as well as an RNA helicase A (RHA), have been implicated in HIV-1 replication, imparting their normal functioning of Rev, specifically the DDX3 [20]–[22]. However, evidence of DDX3 involvement in HIV infection in astrocytes has not yet been established. DDX3, an ATP-dependent RNA helicase [21], functions as a cellular co-factor for CRM1-dependent nuclear export of HIV-1 RNA. DDX3 upon binding to mRNA in the nucleus becomes involved in mRNA translation and transportation to the cytoplasm [23]. In addition, double-strand RNA binding protein, an RNA helicase A (RHA), binds to the TAR element of HIV-LTR and regulates HIV-1 mRNA expression [24]. Substitution of glutamic acid with lysine at position 236 in RHA results in low expression of HIV-1, while overexpression of RHA increases viral replication [24]. Another double-strand RNA binding protein, TRBP, a TAR-binding protein involved in inhibiting PKR activation and a component of the miRNA processing machinery, is under expressed in astrocytes [25]–[28]. It has been suggested that natural under expression of TRBP in astrocytes is responsible for restricted HIV-1 replication. Ectopic TRBP supplementation in astrocytes, which has been found to result in normalization of HIV-1 replication, is thought to occur through inhibition of PKR activation [29]. Apart from its direct activation effect, TRBP reverses PKR-induced suppression of HIV-1 LTR promoter activity [30],[31].

Regulatory Tat protein dramatically increases HIV-LTR-directed transcriptional processing. It does so by binding to the LTR-encoded TAR element of nascent mRNA downstream of the transcription start site upon involvement of several host factors [32]–[36]. Moreover, pleotropic Tat increases the overall level of viral mRNAs, possibly at several different levels, such as involvement of proteasome complex at the promoter region [37],[38], reorganization of chromatin, and induction of several other factors, including suppression of RNAi [39]–[45]. Tat and its cellular co-activator, the positive transcription elongation factor b (p-TEF-b), binds TAR, thus engaging CDK9 and cyclin-T1. This results in hyperphosphorylation of c-terminal domain (CTD) of RNAPII and thus leads to productive elongation of mRNA transcripts [46]–[51]. It is unclear whether critically low expression of Tat protein in naturally infected astrocytes is responsible for restricted viral replication. Several studies have shown crippled HIV Rev function in astrocyte cell lines, which does not completely resemble primary astrocytes. Thus, we examined the regulation of HIV-1 replication in primary astrocytes (HFA) and astrocytic cells (SVGA) derived from fetal astrocytes after SV40 antigen transformation.

RNA helicases, including DDX3, TRBP [26],[29],[52], Sam68 [52]–[56], and hematopoietic cell-specific Lyn substrate 1-associated protein X-1 (Hax-1) [57] in HIV permissive cells have been described as mediators of viral replication and RNA transport [24],[58], we also investigated whether DDX3 and TRBP in astrocytes are involved in Rev function and HIV replication. We found that HIV-1 Tat and Rev functions were unperturbed, leading to persistent infection in astrocytes.

## Results

### Restricted HIV-1 infection in astrocytes

**Figure 1:**
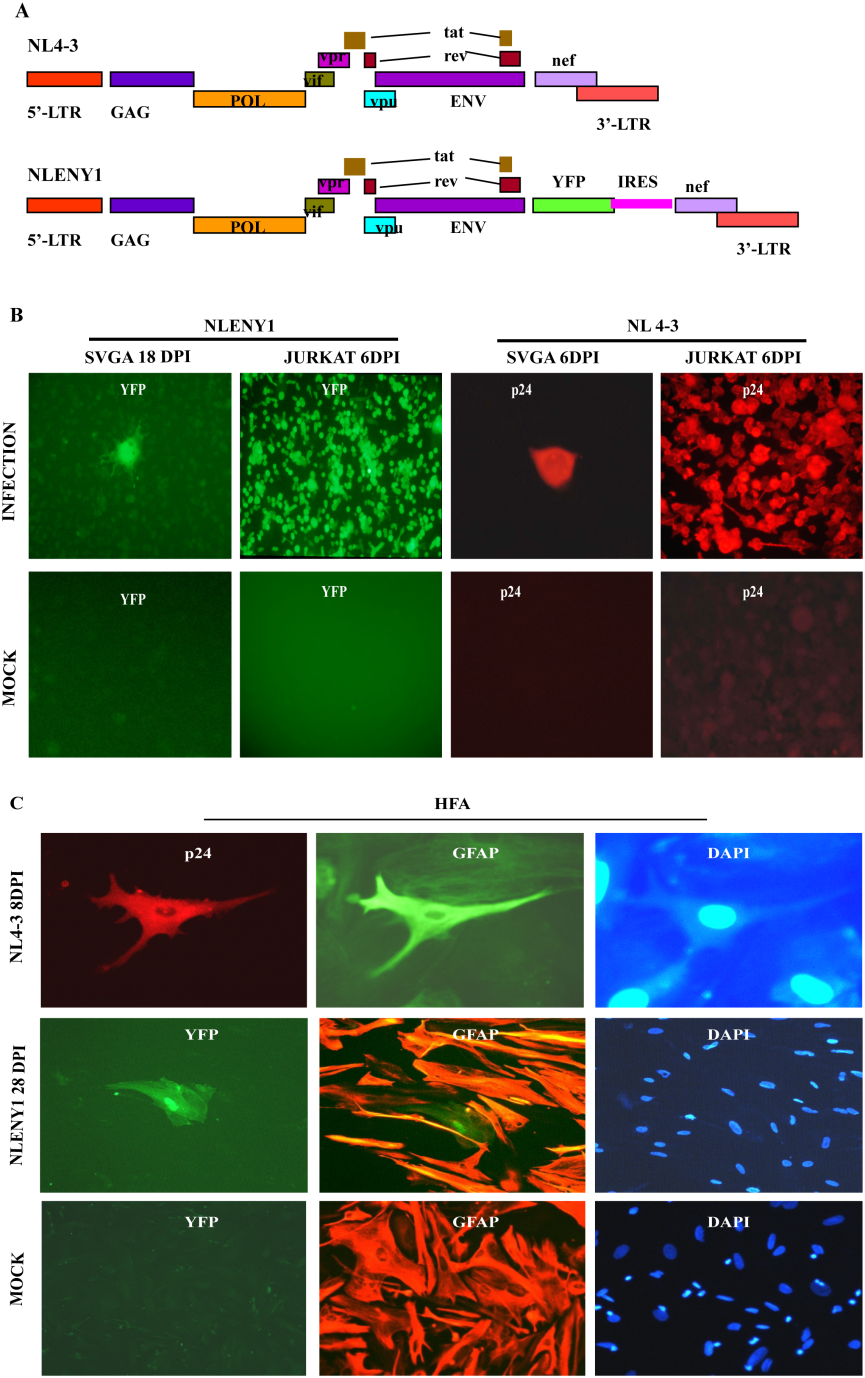
HIV-1 infection in astrocytes. HIV-1 NL4-3 or NLENY1 infection was done in lymphocytes (Jurkat), primary human fetal astrocytes (HFA), and SVGA cells. (**A**) Diagrammatic view of HIV-1 viruses, NL4-3 (T-tropic) wild type, and recombinant NLENY1 genetically engineered NL4-3 showing YFP gene insertion. (**B**) SVGA or Jurkat cells in 6-well culture plates were infected with NLENY1 or NL4-3 virus. After 6–18 days of infection, cells were fixed and directly examined for green fluorescence or immunostained for viral p24 (**Fig. 1B**). Mock-infected SVGA, HFA and lymphocytes served as negative controls for p24 antibody. (**C**) HFA in 60-mm dishes were infected with HIV-1 NL4-3 or NLENY1 for 8–28 days, then immunostained for p24 and GFAP, then examined by UV microscope. Upper panels show NL4-3-infected GFAP-positive astrocytes 8 days after infection; middle panels show NLENY1 infection in HFA on 28 days; bottom panels show mock-infected GFAP-positive HFA as control.

In the current study, primary human fetal astrocytes (HFA) and astrocytic cells (SVGA) were infected with fluorescent reporter HIV-1 NL4-3 (NLENY1) (**Fig. 1A**) [7],[59],[60]. NLENY1 faithfully expresses yellow fluorescent protein upon HIV-1 replication, as described earlier [8],[60]. Experiments were also done with wild type HIV-1 NL4-3 (**Fig. 1A**) on HFA and SVGA [18]. HIV-1 infection was demonstrated by immunostaining for viral structural protein gag (p24) or directly visualizing YFP (green) fluorescence in infected cells. In a positive control, highly productive HIV-1 infection occurred in Jurkat cells (**Fig. 1B**), but minimal infection by recombinant NLENY1 or wild-type NL4-3 viruses was observed in astrocytes (HFA and SVGA) (**Fig. 1B, C**). HIV-1 infection in SVGA and HFA was consistently minute in more than 10 experiments. Authentic HIV-1 infection in astrocytes was detectable only by fluorescent microscopy. In our earlier studies, HIV-1 infection in HFA and SVGA had also minimal infection [8], suggesting perturbed function of early viral regulatory proteins (Tat and Rev) or viral entry restrictions. Thus, to determine whether HIV-1 infection was restricted at the intracellular level by limited functioning of early viral regulatory proteins, investigations were done for the transcriptional or posttranscriptional activities of Tat and Rev in astrocytes, using viral LTR reporters.

### Tat-mediated HIV-1 LTR transcriptional processivity in astrocytes

**Figure 2:**
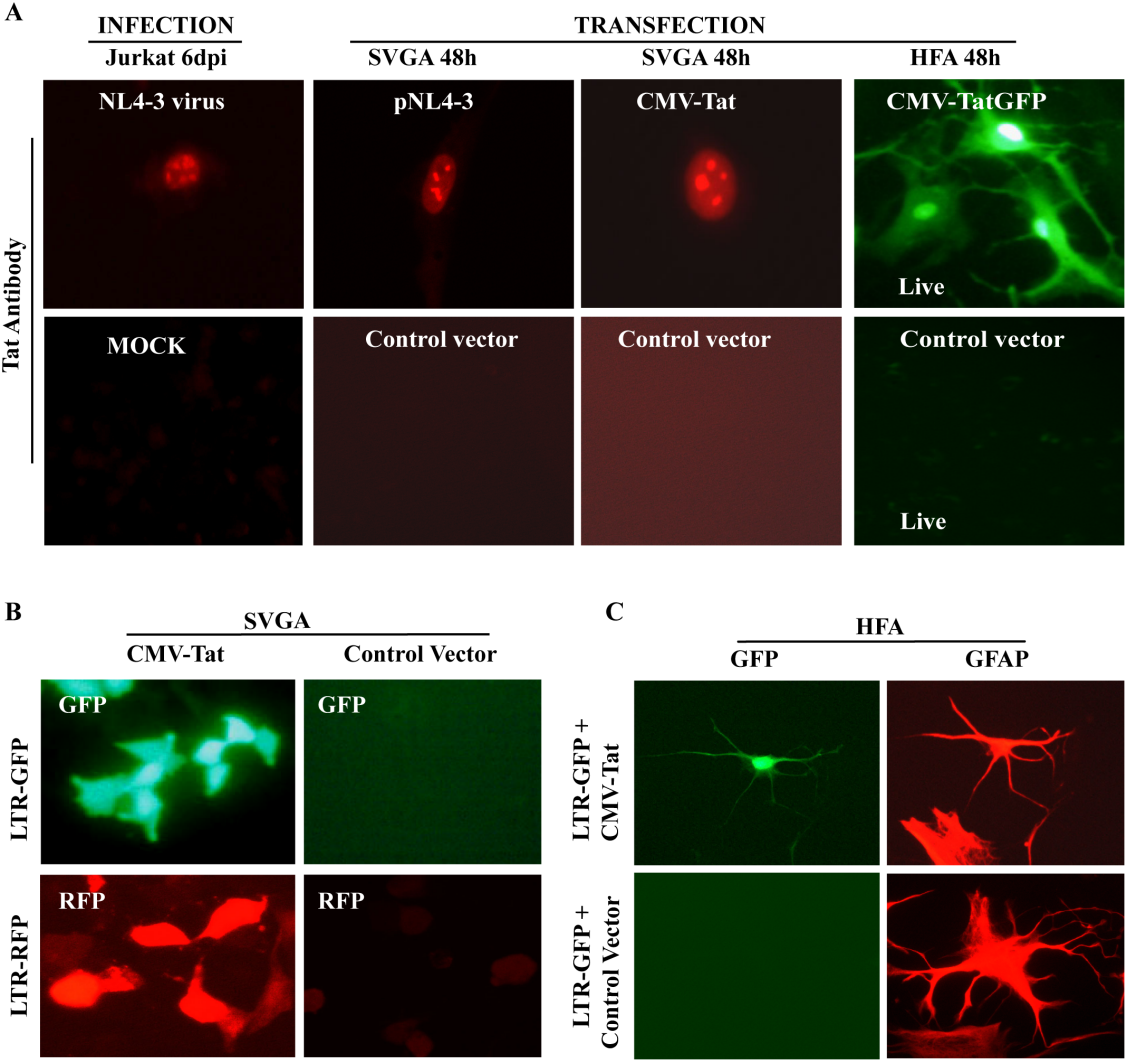
HIV-1 Tat protein expression and its biological activity in astrocytes. **(A)** Jurkat or SVGA were cultured in 6-well culture plates. In one set, cultures were infected with NL4-3 and assessed for Tat expression. HIV-1 NL4-3-infected Jurkat cells at 6 days after infection, pNL4-3 or CMV-Tat expression vector transfected SVGA were immunostained for Tat. In immunostaining, uninfected Jurkat or empty-vector transfected SVGA served as controls for Tat antibody. HFA after 48 h of transfection with Tat-GFP expression vector, were observed by fluorescent microscopy. **(B)** For Tat-mediated HIV-1 LTR transactivation in astrocytes, SVGA reporter cells (LTR-GFP or LTR-RFP) were transfected with Tat expression vector or HFA were cotransfected with Tat-expressing and LTR-GFP vectors. At 48 h after transfection, HFA were immunostained for GFAP (red); fluorescence was observed by UV microscope.

**Figure 3:**
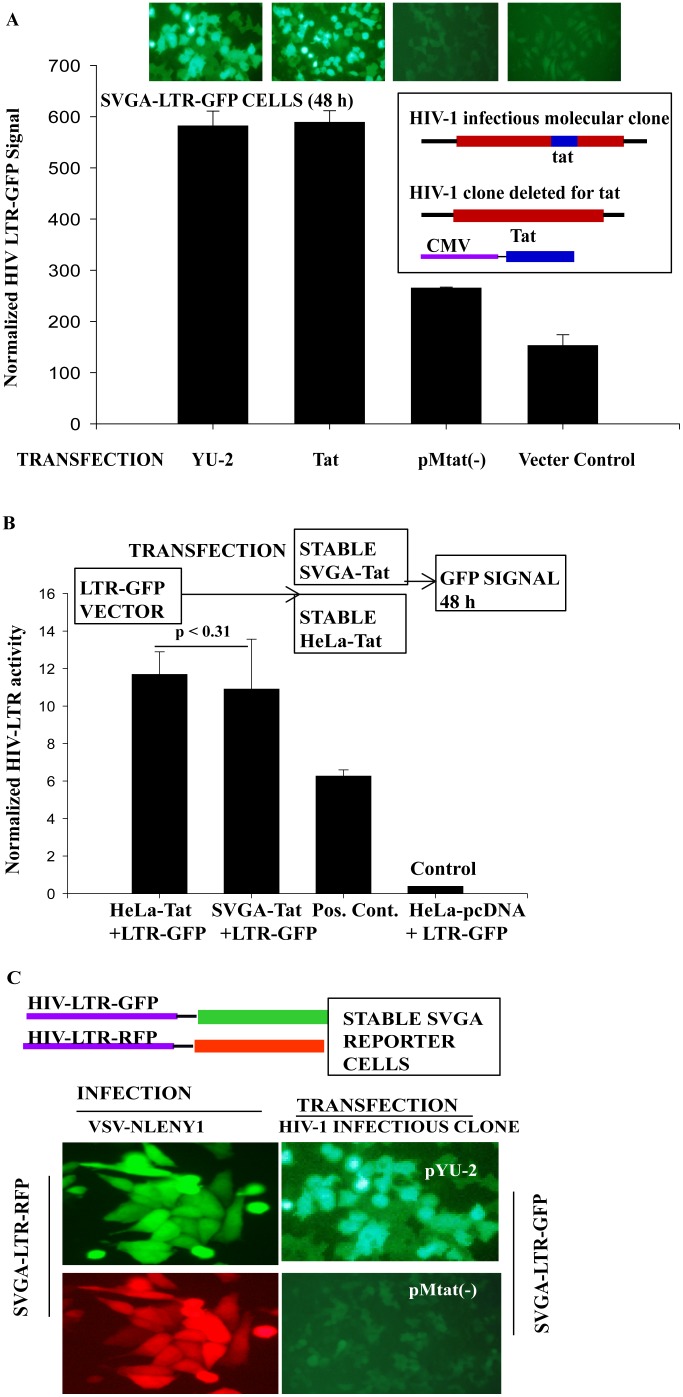
Tat-mediated HIV-1 LTR transcriptional regulation in astrocytes. (**A**) HIV-1 wild-type or mutant infectious molecular clones and Tat expression vector are shown (**Fig. 3A** **inset**). SVGA-LTR-GFP (stable) cells were transfected with 1.0 µg of HIV-1 YU-2 molecular clone, pMtat(-), Tat vector, or pcDNA empty vector. LTR-GFP fluorescence was monitored 48 h later by UV microscope or read in a fluorimeter. (**B**) Stable HeLa or SVGA cells expressing minimal levels of Tat (undetectable by immunostaining) were transfected with 0.5 µg of LTRGFP plasmid. SVGA-pcDNA (stable) cells transfected with LTR-GFP vector served as controls. HIV-LTR activity in SVGA-Tat was comparable to that in HeLa-Tat cells (p≤0.31; n = 2). (**C**) Stable SVGA reporter cells (LTR-GFP or LTR-RFP) were infected with VSV-NLENY1 or transfected with HIV-1 YU-2 infectious molecular clone or HIV-1 infectious molecular clone mutated for tat gene pMtat(-). Red (Tat-mediated) and green fluorescence (HIV-1 infection) was observed 48 h later by UV microscope.

We first investigated the expression pattern of Tat in astrocytes and HIV-1 permissive lymphocytes. As in earlier studies [61],[62], immunofluorescence showed Tat in the nuclei and nucleoli of HIV-1 infected lymphocytes (**Fig. 2A**) and ectopically Tat-expressing astrocytes, but not in controls (**Fig. 2A**). We also examined Tat-mediated transcriptional regulation of HIV-1 LTR in SVGA and HFA. Co-transfection of HIV-1 LTR reporter and Tat expression vectors in SVGA and HFA resulted in robust transactivation of LTR-GFP or LTR-RFP, but not empty vector control (**Figs. 2B, C**), indicating that HIV-1 LTR promoter operates optimally in astrocytes. HIV-1 DNA in natural virus-infected cells is integrated into host DNA. To mimic this situation, we developed stable SVGA-LTR-RFP or LTR-GFP reporter cells. We did transfection studies to verify that stable HIV-1 LTR astrocytic reporter cells could be reactivated by either HIV-1 infectious molecular clone or Tat expression vector. As expected, strong LTR transactivation signals in stable astrocytic reporter cells were achieved by transfecting HIV-1 infectious molecular clone or Tat expression vectors, but not Tat-mutated HIV-1 infectious molecular clone pMtat(-) or control vector (**Fig.** **3A**).

Assuming that minimal levels of Tat are naturally expressed in HIV-1-infected astrocytes and to determine whether these concentrations can activate viral LTR, we developed stable low-Tat-expressing SVGA and HeLa cells [61]. Transient transfection of HIV-1 LTR-GFP construct demonstrated robust LTR transactivation signals in stable Tat-expressing SVGA and HeLa cells, but not in Tat-deficient cells (**Fig. 3B**). Co-transfection of Tat-expressing vector with LTR-GFP vector in SVGA was used as a positive control. There was no significant difference in LTR activity in Tat-SVGA and -HeLa cells (p≤0.31; n = 2) (**Fig. 3B**). These results therefore suggest that minimally expressed Tat in astrocytes efficiently regulated LTR promoter. To corroborate transfection-based Tat functional activity, we infected SVGA-LTR-RFP reporter cells with VSV-NLENY1 virus expressing YFP. Expression of YFP and RFP fluorescence was robust in HIV-1 infected reporter astrocytes. RFP fluorescence indicated Tat expression after infection (**Fig. 3C**).

### Posttranscriptional regulatory function of Rev protein in astrocytes

**Figure 4:**
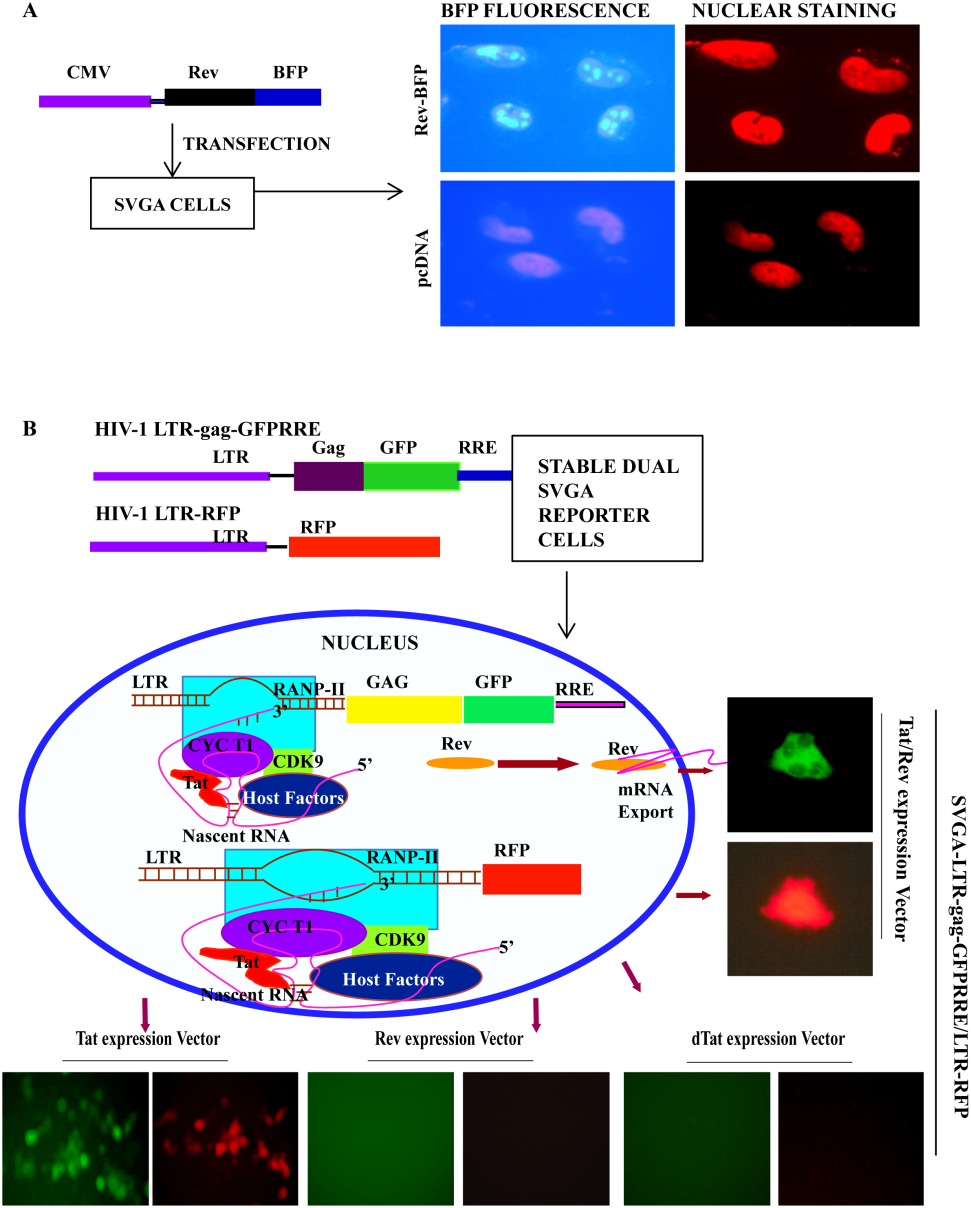
HIV-1 Rev protein expression and its biological activity in astrocytes. (**A**) SVGA cells were seeded overnight in 6-well culture plates, then transfected with Rev-BFP expression vector or empty vector (control). After 24 h, cultures were fixed and their nuclei stained with propidium iodide. Fluorescence was observed by UV microscope. (**B**) Rev- and Tat-responsive reporters and their regulation by Rev and Tat. Stable single and dual reporter expression vectors are shown. SVGA-LTR-gag-GFPRRE/LTR-RFP cells were seeded overnight in 6-well culture plates, then transfected with Tat, Rev, dTat (control), or Tat+Rev expression vector (pCV1); 24 h later, Tat- and Rev-mediated green and red fluorescence signals were observed by UV microscope.

**Figure 5:**
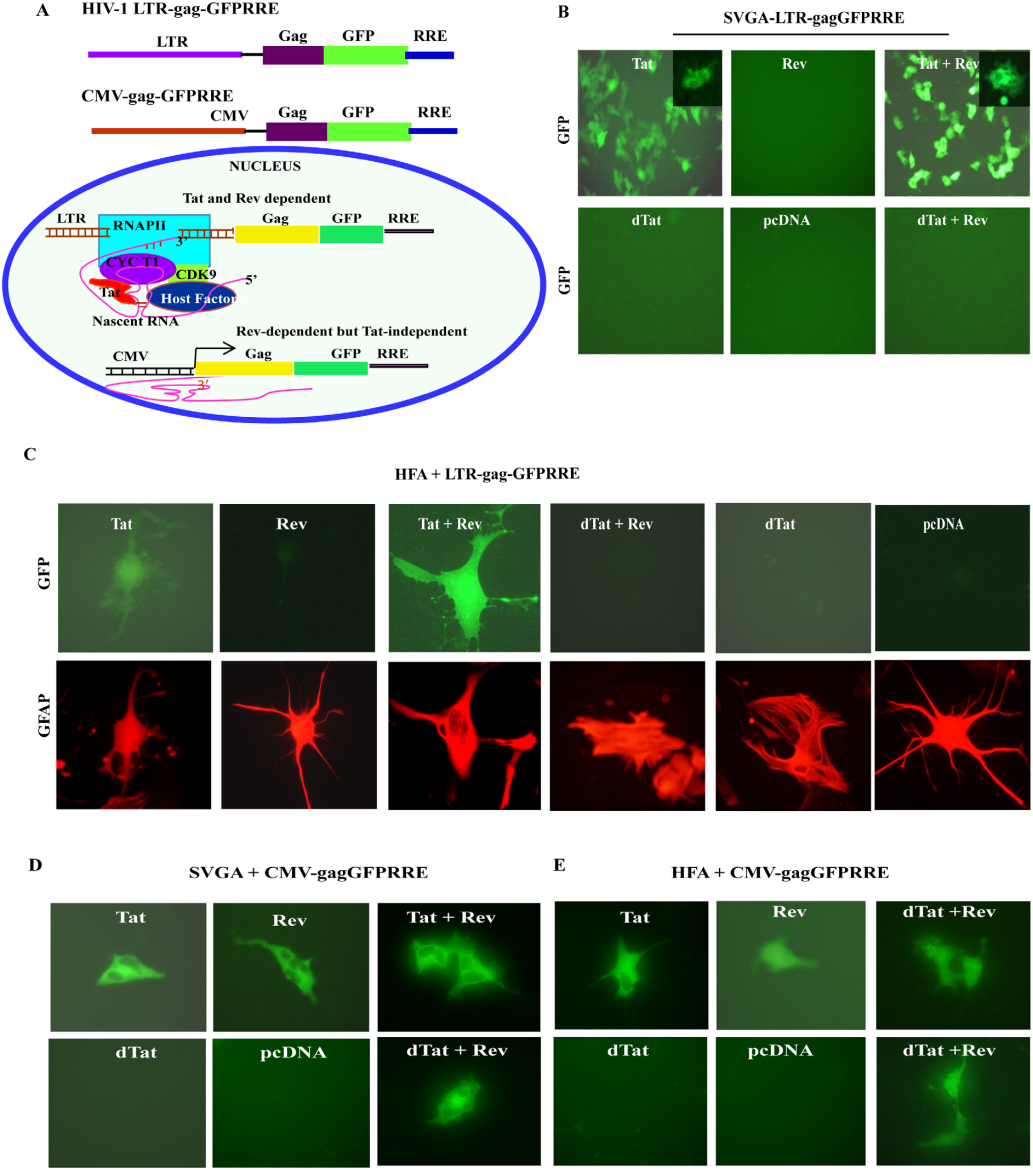
Posttranscriptional regulatory function of HIV-1 Rev in astrocytes. (**A**) Diagram of Tat- and Rev-responsive or Tat-independent but Rev-dependent reporter vectors and their functioning. (**B**) SVGA-LTR-gag-GFPRRE cells seeded overnight in 6-well culture plates were transfected in duplicate with either Tat, Rev, Tat+Rev, dTat, pcDNA (control), or dTat +Rev vectors. GFP fluorescence was captured 48 h later using a digital camera. (**C**) HFA were seeded overnight in 6-well culture plates. The next day, HFA were co-transfected in duplicate with HIV-1 LTR gagGFPRRE and either Tat, Rev, Tat+Rev, dTat+Rev, dTat, or pcDNA (control). Astrocytes were fixed 48 h later and immunostained for GFAP. (**D**) SVGA or (**E**) HFA seeded in 6-well culture plates were co-transfected in duplicate with CMV-gagGFPRRE and either Tat, Rev, Tat+Rev, dTat, pcDNA, or dTat+Rev. GFP fluorescence was captured 48 h later.

Since Rev has been the center of focus with respect to restricted HIV-1 infection in astrocytes [22],[63],[64], was investigated for its posttranscriptional activity. Rev shuttles between the nucleus and cytoplasm, transporting RRE containing unspliced (gag-pol, HIV genomic RNA), or partially spliced HIV transcripts (Env, Vif, Vpr and Vpu) to the cytoplasm [12]. To investigate Rev functioning, studies on HFA and SVGA cultures were conducted. Upon plasmid-mediated Rev expression in astrocytes, Rev was localized to nuclei (**Fig. 4A**). Sensitive and efficient single (SVGA-LTR-gag-GFPRRE) [18] or dual (SVGA-LTR-gag-GFPRRE and LTR-RFP) fluorescent reporter systems (see **SI**) were used to examine Rev function in astrocytes (**Figs. 4B** **,** **5**). The functional activity of Tat and Rev upon transfection of Tat and Rev expression vectors was confirmed in dual reporter SVGALTR-RFP/LTR-gag-GFPRRE (**Fig. 4B**) or single reporter SVGA-LTR-gag-GFPRRE cells (**Fig. 5A**). As expected, vectors expressing Rev or dTat (mutant of Tat) alone did not produce fluorescence in reporter assays. Tat vector alone not only produced LTR-driven red fluorescence, as expected, but also green fluorescence, which was not expected (**Fig. 4B** **,** left bottom panel). Tat and Rev functions were examined in more detail in SVGA reporter cells and HFA.

Vectors encoding Tat, Rev, Tat+Rev, dTat+Rev, dTat, or pcDNA were transfected into SVGA-LTR-gag-GFPRRE reporter cells (**Fig. 5A**); 48 h later, green fluorescence was recorded. Tat and Tat+Rev expression led to a green fluorescence signal in reporter cells, but not Rev, dTat, or control vector (pcDNA) (**Fig. 5B**). The effect of Tat was again unexpected and was assumed to be a transcriptional effect on HIV-LTR and, possibly, the transport of excess mRNA from the nucleus to the cytoplasm by an unknown mechanism. To validate the Rev and Tat function in primary astrocytes (HFA), we did Rev-responsive reporter-based studies. Co-transfection in HFA with LTR-gag-GFPRRE and Tat, Rev, Tat+Rev, dTat+Rev, dTat, or pcDNA vectors resulted in efficient gag-GFP expression in GFAP-positive astrocytes by Tat and Tat+Rev, but not Rev alone or dTat+Rev, dTat, or pcDNA (**Fig. 5C**).

To confirm the involvement of Tat in nuclear export of viral mRNAs independent of the viral LTR-promoter effect, we constructed CMV promoter-driven gag-GFPRRE (CMV-gag-GFPRRE) reporter, in which GFP mRNA transcription is no longer driven by HIV-LTR (**Fig. 5A**). This construct is Tat-independent in transcription, but Rev-dependent for mRNA translation (**Fig. 5A, D**). To test the hypothesis, SVGA cells were co-transfected with CMV-gag-GFPRRE and Tat, Rev, Tat+Rev, dTat+Rev, dTat, or pcDNA vectors and 48 h later, recorded green fluorescence. As expected Tat, Rev, Tat+Rev and dTat+Rev expression led to GFP fluorescence, but not to dTat or pcDNA vectors. Again, the Tat effect on mRNA export was consistent even when gag-GFP was transcribed under CMV-promoter (**Fig. 5D**). To corroborate the results on HFA, CMV gag-GFPRRE construct was transfected with Tat, Rev, Tat+Rev, dTat+Rev, dTat, or pcDNA vectors. As with SVGA, HFA-transfected cultures 48 h later showed green fluorescence in Tat, Rev, Tat+Rev and dTat+Rev, but not dTat or pcDNA vectors (**Fig. 5E**).

**Figure 6:**
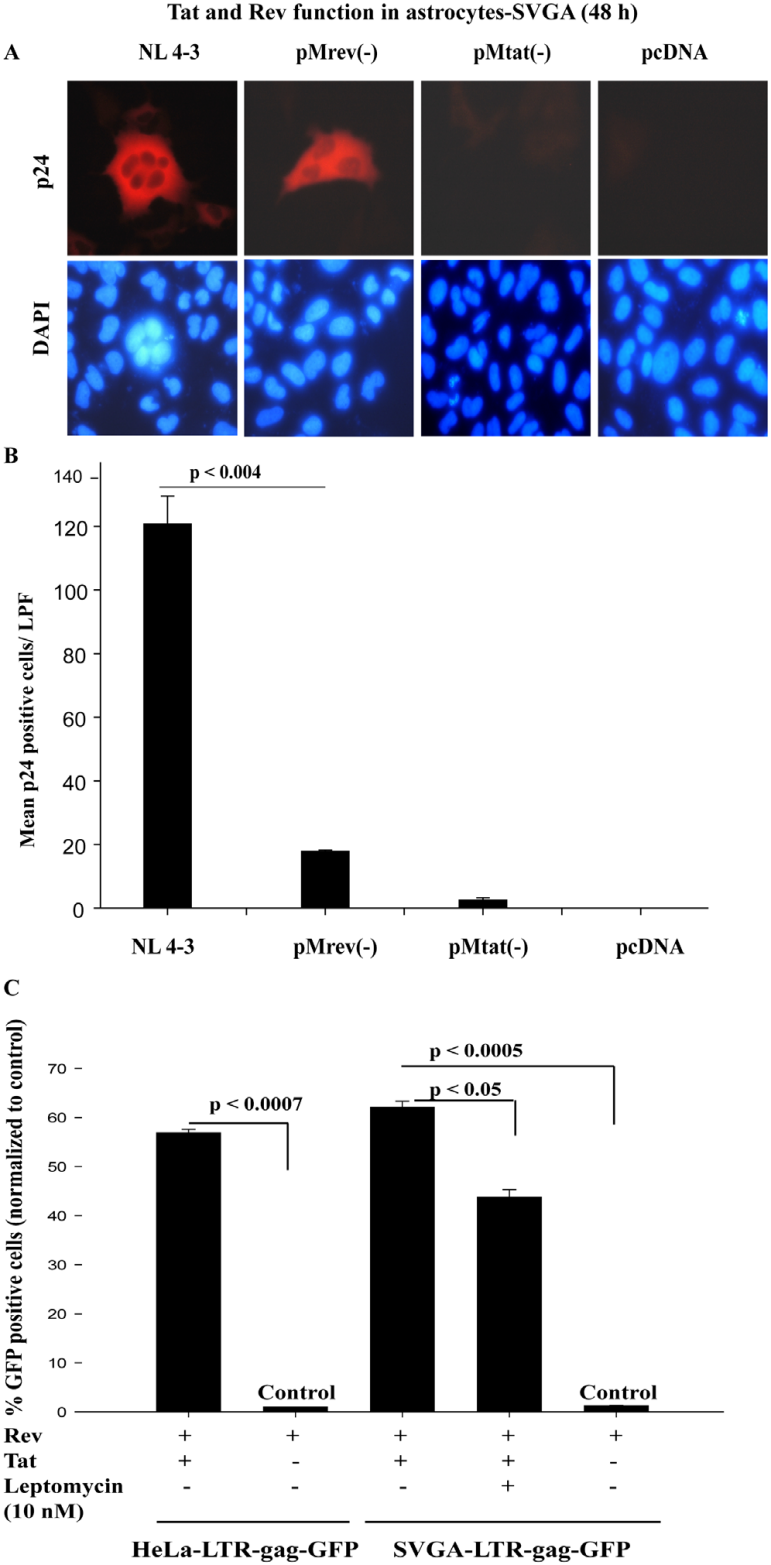
HIV Rev predominantly regulates the posttranscriptional viral life gene expression in astrocytes. SVGA seeded in 6-well culture plates were transfected in duplicate with full length HIV-1 infectious DNA clone, HIV DNA mutated for Rev pMrev(-), HIV DNA mutated for Tat pMtat(-) or pcDNA vector as control. Cells were fixed 48 h later and immunostained for HIV gag (p24) and nuclei. The immunostained cells were photographed (**A**) and counted (**B**) for p24 positive cells in ten random fields under low power field (LPF) in duplicate sets. Means were plotted and p values calculated using t test (p<0.004) (n = 3). (**C**) HIV-1 LTR-gagGFPRRE activity in SVGA and Hela cells: Stable SVGA-LTRgagGFPRRE and Hela-LTRgagGFPRRE cells were seeded overnight in 12-well culture plates. On the next day, reporter cells were transfected with Tat−Rev, Rev, or control pcDNA vector. Leptomycin B (10 nM) was used as a positive control to inhibit Rev function. At 72 h after transfection, GFP-positive cells were counted in tests and controls in duplicate sets, then plotted (p<0.0007; p<0.0005) (n = 2).

These observations, based on more than five independent experiments on SVGA and HFA with rigorous controls, indicated the role of Tat in post-transcriptional regulation of HIV-1 gene expression was not a promoter-based difference in reporter assays (HIV-LTR versus CMV). Further studies were designed to investigate Tat function. HIV-LTR-gag-GFPRRE and CMV-gag-GFPRRE reporters do not absolutely mimic viral RNA splicing and nuclear export. Given that and to have a proof of concept, we validated Tat and Rev functions in astrocytes using full length HIV-1 infectious DNA (pNL4-3) and its mutants of Tat pMtat(-) and Rev pMrev(-). Further experiments were performed in SVGA cells only because transfection efficiency is far greater in SVGA than in HFA and to obtain conclusive data. pNL4-3, pMrev(-) mutated for Rev, but expresses Tat and other viral proteins, pMtat(-) mutated for Tat gene and pcDNA control vectors were transfected in SVGA. At 48 h after transfection, cells were immunostained for gag (p24) and nuclei (**Fig. 6A**). p24 positive cells were counted in 10 random fields in duplicate sets under low-power field (LPF). Means were plotted (**Fig. 6B**) and analyzed by t-test (p<0.004). p24 expression was robust in pNL4-3, sporadic in pMrev(-), but absent in pMtat(-) and control vector transfected astrocytes (**Fig. 6B**). Experiments repeated three times yielded these results consistently. Hence, Rev is mainly involved in nuclear export of HIV mRNA, but Tat independently also plays a minor role.

Rev function in SVGA was also quantitatively compared to HIV-permissive HeLa cells. On Tat and Rev expression, HeLa- and SVGA-LTR-gagGFP reporter cells had similar Rev-mediated gag-GFP signals (**Fig. 6C**), which were inhibited by leptomycin B (see **SI**). This indicated specificity and normal functioning of Rev in astrocytes. Overall, our studies indicated that the regulatory activities of HIV-1 Tat and Rev proteins in primary astrocytes and astrocytic cells were not only unperturbed, but comparable to those of HIV permissive HeLa cells.

### RNA helicase DDX3 regulates HIV-1 Rev function in astrocytes

**Figure 7:**
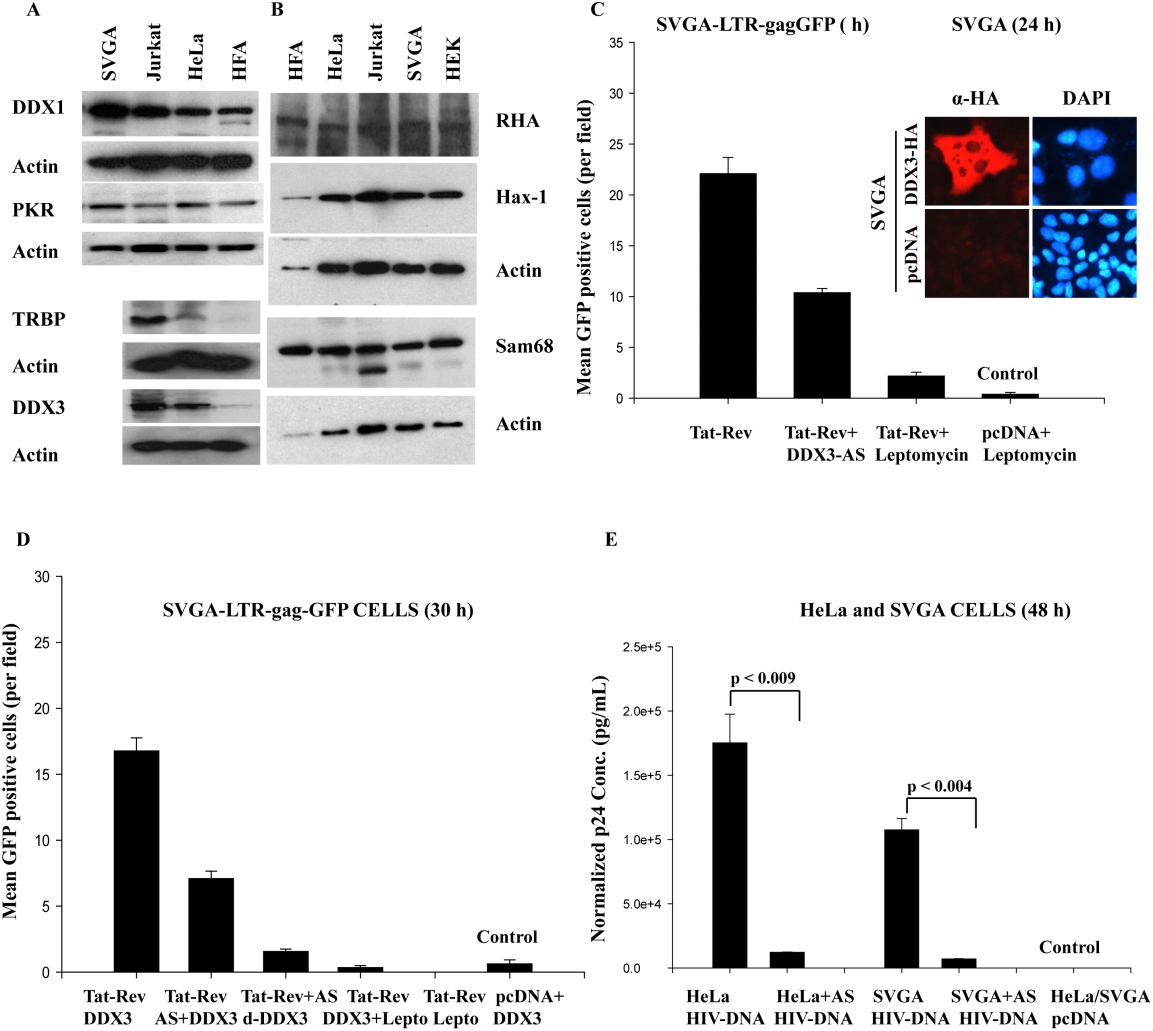
RNA helicase DDX3 regulates HIV-1 Rev function in astrocytes. (**A–B**) Astrocytes (HFA and SVGA) and HIV-1-permissive cells (Jurkat, HEK, and HeLa) were cultured in 6-well culture plates, and 24 h later, lysed and processed for Western blotting. Natural expression levels of RNA helicases (DDX1, DDX3, and RHA), HAX-1, Sam68, PKR, and TRBP in SVGA and HFA are shown for comparison with HIV-1-permissive cells. (**C**) SVGA were cultured in 6-well culture plates and, 24 h later, transfected with DDX3-HA expression vector or control vector. After 48 h, immunostaining for DDX3 was done in transiently transfected astrocytes using HA tag antibody (**inset**). In another set, SVGA-LTR-gagGFP cells were seeded in 12-well culture plates and transfected with Tat+Rev, Tat+Rev+antisense-DDX3 (AS), or pcDNA vector (control). Leptomycin-B was used a positive control for inhibition of Rev function. (**D**) Ectopic DDX3 expression partially reinstated AS-ablated Rev function. Cultured SVGA-LTR-gagGFP reporter cells in 12-well plates were transfected with Tat+Rev expression vector alone or in combination with DDX3, AS, or mutant DDX3 (d-DDX3, functionally dead mutant). In parallel, reporter cells were transfected with control empty vector. Leptomycin B was used as a positive control for inhibition of Rev function. At 30 h after transfection, GFP-positive cells were counted in 10 random fields in duplicate sets and plotted. (**E**) HeLa and SVGA cells in duplicate were transfected with HIV-1 NL4-3 infectious molecular clone (pNL4-3) alone or in combination with AS or d-DDX3 expression vectors. In parallel, cells were transfected with empty vector (control). At 48 h after transfection, p24 in supernatants was monitored in triplicate by ELISA (p<0.004 and p<0.009) (n = 2).

Our investigations so far have shown restricted HIV-1 infection in astrocytes inspite of their having normal Tat and Rev regulatory activities. Further, multiple factors such as Sam68, TRBP, and PKR, have been implicated in restricted HIV-1 infection in astrocytes. To implicate their involvement in HIV-1 infection, studies were conducted in astrocytes. Further, DEAD box RNA helicase, DDX3, has also been shown to be involved in Rev function, but so far no studies have been done on astrocytes. To corroborate our data, we examined whether the RNA helicases DDX1, DDX3, and RNA helicase A (RHA) are required for optimum functioning of Rev in HIV-1-infected astrocytes. Initial protein profiling of astrocytes (HFA and SVGA) and HIV-1-permissive cells by Western blotting showed comparable levels of DDX1 and RHA, but barely detectable levels of DDX3 in HFA as compared to levels in other cells (**Fig. 7A, B**). It has been reported that under-expression of TRBP [29] and Sam-68 [56], as well as natural expression of PKR [29] and Hax-1 proteins, severely cripple the HIV-1 life cycle in astrocytes. To investigate these proteins, we first monitored their cellular expression levels. Western blotting demonstrated comparable levels of Sam 68, PKR, and Hax 1 proteins, but TRBP, was barely detectable in HFA as compared to other HIV-1-permissive cells, including astrocytic (SVGA) cells (**Fig. 7A, B**).

We investigated DDX3, TRBP, and PKR in greater detail, finding that, on transient transfection of DDX3 vector in astrocytes, expression occurred mainly in the cytoplasm and only rarely in nuclei (**Fig. 7C** **inset**). To elucidate the role of DDX-3 in HIV Rev function, we ablated endogenous DDX3 mRNA translation, using an antisense-DDX3 (AS) strategy and, as a control, leptomycin B, an inhibitor of Rev function. AS-DDX3 severely impaired Rev-dependent reporter expression (gag-GFP) in SVGA-LTR-gagGFPRRE cells (**Fig. 7C**). AS-mediated inhibition of Rev function was partially rescued by overexpression of DDX3, but not by mutant DDX-3 (d-DDX3) (**Fig. 7D**). In controls, transfection of SVGA-LTR-gag-GFP reporter cells with Rev and Tat expression vectors, followed by treatment with leptomycin-B, strongly inhibited Rev function (**Fig. 7C, D**). However, leptomycin B-mediated inhibition of Rev-activity could not be reversed by DDX3-overexpression (**Fig. 7D**).

To strengthen these findings, we overexpressed AS in pNL4-3 DNA-transfected astrocytes and HeLa cells, then monitored viral production by p24 in culture supernatants (ELISA). As compared to control vector, AS-mediated ablation of DDX-3 profoundly crippled viral production (p24) (**Fig. 7E**, p≤0.009, p≤0.004). Thus, Rev requires DDX3 for normal HIV-1 replication in astrocytes. We suspected that natural under-expression of DDX3 or TRBP could be responsible for low replication of HIV-1 in astrocytes. Although natural levels of DDX3 and TRBP were barely detectable in HFA, these were adequate to perform normal HIV-1 replication (**Fig. 7**).

### HIV-1 entry barrier bypass in astrocytes leads to robust Rev-dependent viral replication

**Figure 8:**
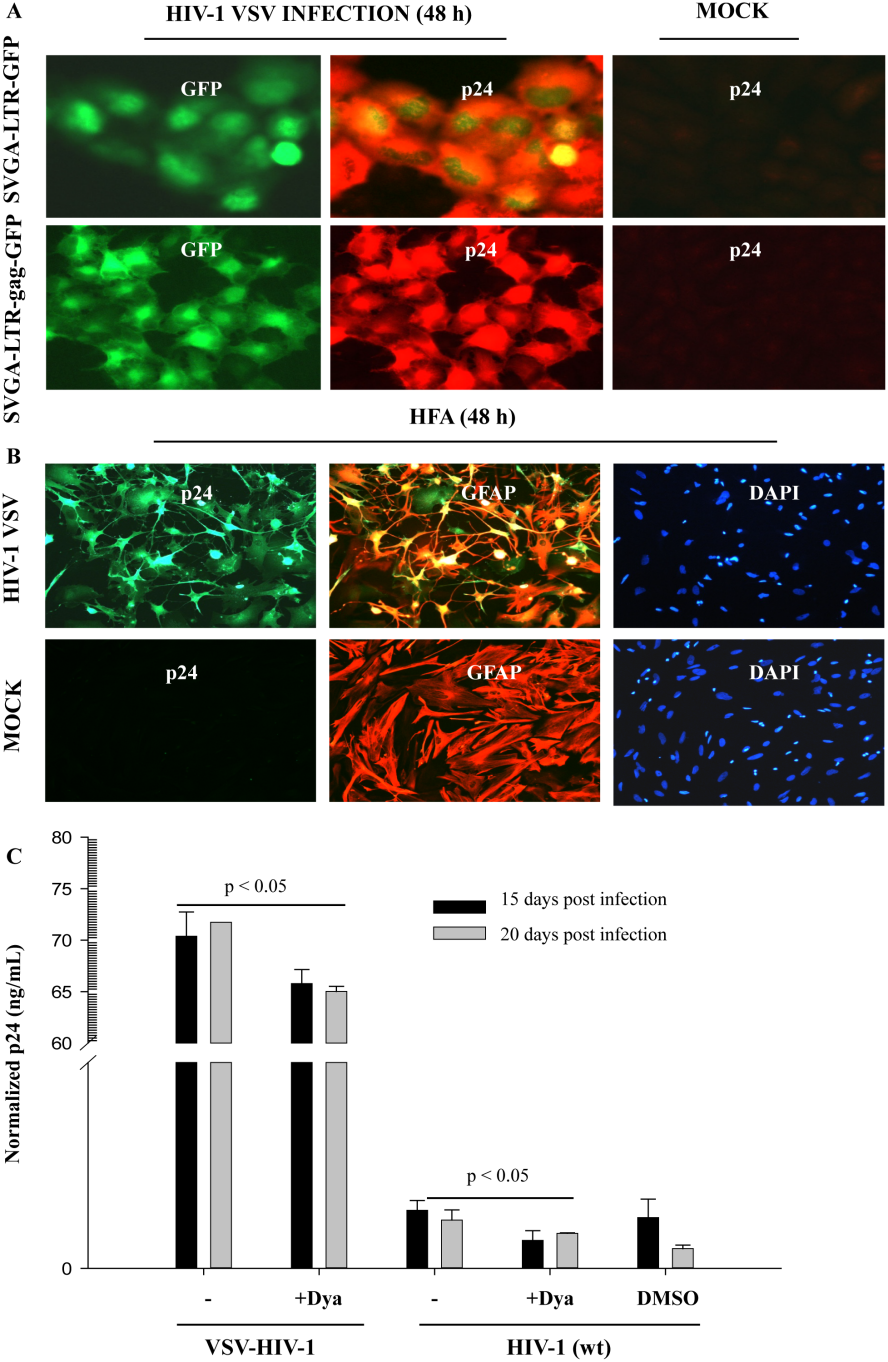
HIV-1 entry bypass in astrocytes and Rev function. (**A**) SVGA-LTR-GFP and SVGA-LTR-gagGFP reporter cells were seeded in 6-well culture plates. Reporter cells were infected with VSV-NL4-3. At 48 h, infected cells were fixed and immunostained for p24. Uninfected cells served as controls for p24 antibody. Immunostained specimens were observed by UV microscope and photographed. (**B**) HFA in flaskets were infected with VSV-NL4-3 and 48 h later immunostained for p24 and GFAP. Uninfected HFA served as a control for p24 antibody. After immunostaining, nuclei were stained with DAPI and examined by UV microscope. (**C**) HFA cultured overnight in 6-well culture plates were left untreated or treated with Dynasore. HFA cultures were infected with VSV-NL4-3 or NL4-3 virus. In parallel, uninfected cultures were used as controls. HFA cultures were followed for 20 days, after which the supernatants were analyzed for p24 (ELISA), normalized with normal controls, and plotted (p<0.05; p<0.05) (n = 3).

To corroborate transfection-based Rev function and overcome viral entry restrictions, we established a robust model of HIV-1 infection in astrocytes using VSV-pseudotyped HIV-1. We infected SVGA-LTR-GFP or LTR-gag-GFPRRE reporter cells with VSV-pseudotyped NL-4-3 virus. In both reporter systems, VSV-pseudotyped NL4-3 virus infection produced strong Tat- and Rev-dependent signals (GFP and gag-GFP), leading to high expression of viral capsid protein p24 (**Fig. 8A**). Mock-infected (control) cells did not show p24 expression.

Validating these findings on primary astrocytes, VSV-NLENY1 fluorescent virus infection showed profound infection by expressing YFP and viral p24 protein in GFAP-positive astrocytes (**Fig. 8B**). To provide specificity in viral entry experiments, dynamin was used as a control. In our earlier study, VSV-HIV-1 entry was drastically inhibited by Chloroquine and bafilomycin A (see **SI**), suggesting endocytosis-mediated viral entry. However, wild-type HIV-1 infection was bolstered by these lysosomotropic agents [8]. Thus, we used Dynasore (see **SI**), a dynamin inhibitor. Because a study [65] of other cell types has shown that dynamin is involved in HIV-1 entry, we tested whether blocking dynamin prevents HIV-1 entry into astrocytes. We treated HFA with Dynasore, then infected them with VSV-HIV-1 or HIV-1 (wt). Dynasore treatment inhibited both VSV-HIV-1 and wild-type virus infection for upto 20 days of follow-up (**Fig. 8C** **,** p<0.05).

**Figure 9:**
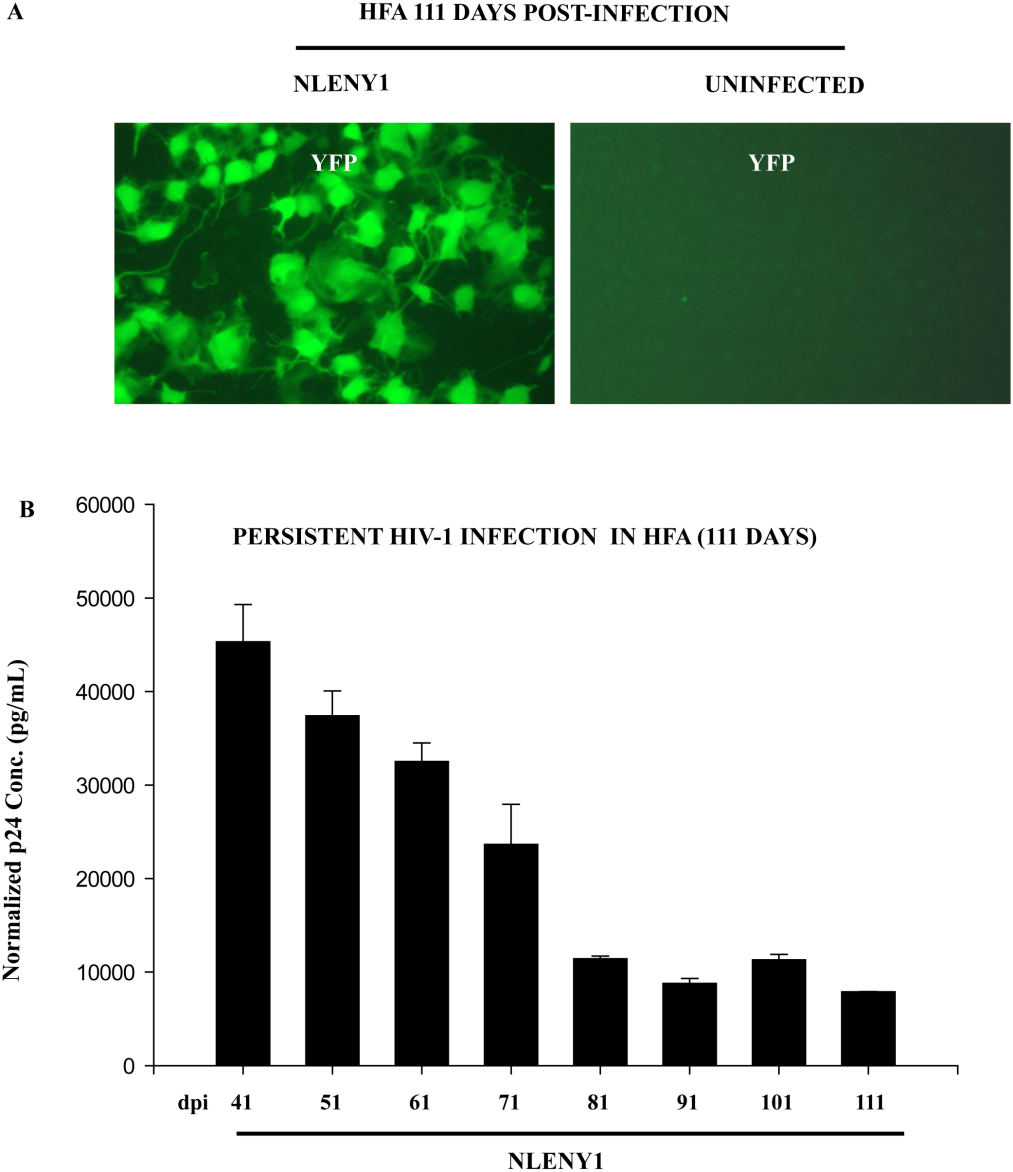
HIV-1 entry bypass in astrocytes leads to persistent infection. HFA seeded in T-25 flasks were infected with VSV-pseudotyped NLENY1 virus, and, 2 h later, washed and cultured at 37°C. Cultures were followed for 111 days. Every 10 days, cultures were examined for green fluorescence (YFP) in cells; culture supernatants were collected for p24 analysis. (**A**) YFP (green) expression at 111 days in HIV-1-infected HFA is shown. (**B**) Viral p24 levels were analyzed after normalization with uninfected controls and plotted (n = 2).

Since VSV-HIV-1 infection in astrocytes remained robust for 20 days, we followed virus-infected HFA from Day 26 through Day 111, using astrocyte-specific YFP (green) fluorescence and viral p24 in infected culture supernatants (ELISA) as markers. We observed robust YFP expression in HIV-1 infected HFA (**Fig. 9A**) and high levels of viral p24 protein in culture supernatants at 111 days (**Fig. 9B**). Extracellular viral p24 levels in HIV-1-infected astrocyte cultures declined sequentially through 111 days, but still remained at high levels (**Figs. 9B**). At 111 days after infection of HFA, Alu-HIV-LTR PCR showed HIV-1 integration [8], suggesting the occurrence of viral replication from an integrated DNA state. Overall, our observations on HIV-1-infected HFA and SVGA cells indicated normal Tat and Rev functions and the persistence of viral infection for several weeks.

## Discussion

Earlier studies showed that HIV-1 infection in astrocytes is unproductive [9],[66],[67]. However, several studies, including ours, have now shown that HIV-1 infection in astrocytes, though minimal, is productive [1],[7],[8],[68]–[70]. Early studies also demonstrated blockage of Rev activity and inefficient translation of viral mRNAs in astrocytes, implicating factors such as Sam-68 [56], PKR, and low levels of TRBP [29]. In the current study, as in our earlier work [7],[8], HIV-1 infection, as a consequence of limited viral entry, was barely productive. Post viral entry restrictions were not observed and Tat- and Rev-functions were unperturbed.

HIV-1 replication in astrocytes is limited by PKR, which binds to the TAR region and inhibits viral mRNA translation as a consequence of naturally low levels of the PKR inhibitor, TRBP [28],[29]. In the current study, as in earlier ones, TRBP was barely detectable in primary astrocytes [29]. However, overexpression of TRBP or inhibition of PKR by siRNA did not enhance HIV-1 replication in astrocytes. An earlier study [21] on HIV-permissive cells elegantly demonstrated the critical role of DDX3, a host RNA helicase, in Rev function and HIV-1 replication. In our analysis, levels of DDX1 and DDX3 proteins in astrocytic cells (SVGA) were comparable to those in HIV permissive cells. In primary astrocytes (HFA), however, DDX3 was barely detectable. Initial studies indicated that limited HIV-1 infection was similar in HFA and SVGA, suggesting that naturally higher levels of DDX3 in SVGA give no advantage. However, inhibition of DDX3 by antisense-DDX-3, Rev activity severely crippled HIV-1 replication in astrocytes. Notably, overexpression of DDX-3 partially restored antisense-DDX-3- inhibited Rev activity, corroborating the critical role of this RNA helicase in HIV-1 replication. HIV-1 infection was unaffected in the presence of barely detectable levels of DDX3 and TRBP, and high levels of antiviral PKR and Hax1 proteins. Neither overexpression of DDX3 and TRBP nor ablation of PKR by siRNA provided any advantage to HIV-1 replication in astrocytes. However, primary astrocytes compared to astrocytic cells barely express DDX3 and TRBP proteins.

Our results on intact Rev activity in astrocytes were corroborated by successful VSV-pseudotyped HIV-1 infection of primary astrocytes and SVGA-reporter cells. Canki et al. [5] have shown efficient production of 9-, 4- and 2-kb viral mRNA transcripts and production of p24 in VSV-pseudotyped HIV-1 infection in astrocytes. These findings were corroborated by our recent study [8]. Intriguingly, the use of recombinant HIV-1 NLENY1 virus expressing yellow fluorescent protein or fluorescent astrocytic reporter cells unambiguously demonstrated barely productive viral infection in astrocytes, which was detectable only by fluorescence microscopy. This infection in astrocytes was far below the limit of the HIV-1 p24 detection assay (ELISA), as we found in an earlier study [8]. It seems that viral entry into astrocytes leads to barely detectable viral infection because, as we have shown, most of the endocytosed virus is retained and degraded in endosomes or proteasomes [7],[8]. In those studies, treatment of HIV-1-infected astrocytes with autophagy, endosomal, or proteasomal inhibitors led to increased viral infection [7],[8], suggesting that wild-type HIV-1 infection in astrocytes is restricted during endocytic viral entry. Further, VSV-HIV-1 entry occurs via endocytosis, while wild-type HIV-1 enters lymphocytes using CD4 and either CXCR4 or CCR5 receptors. Overcoming the bias of using VSV-pseudotyped HIV-1 in our earlier studies, ectopic CD4 expression in primary astrocytes led to robust wild-type HIV-1 infection [8], demonstrating that the intracellular environment in astrocytes is conducive to viral replication. Using Tat and Rev-responsive reporter systems and HIV-1 infectious DNA clones, revealed normal Tat and Rev functions in astrocytes. One unexpected observation was that Tat has a role in posttranscriptional regulation of viral gene expression. However, Tat activity was far less than that of Rev. Tat can regulate the HIV-1 infectious cycle in the presence of crippled Rev function, but only to a limited extent. Transient transfection of HIV-1 infectious molecular clones in astrocytes showed high levels of HIV-1 replication and release of infectious virus particles [13],[14], suggesting, again, a suitable intracellular environment for viral replication.

The use, in our earlier studies, of fluorescent reporter virus infection yielded other interesting observations, such as sustained production of Tat and Rev, which, as we confirmed in this study, led to the persistence of productive infection. HIV-1 infection in HFA was corroborated by viral integration PCR (see Supporting Information S1) and secretion of virus for 111 days of follow-up. This is similar to our earlier observation of productive HIV-1 infection for 160 days (Chauhan et al., 2014). Although studies using VSV-pseudotyped HIV-1 viruses have reported somewhat persistent infection in human astrocytes [5],[13],[14], it has not occurred to the extent that it did in the current study. Thus, natural HIV-1 infection in astrocytes can indeed be productive, but is minimal [7],[8]. Also, inhibition of acidification enhances HIV-1 infectivity [7],[8],[71],[72]. We have demonstrated that dynamin inhibitor has a negative effect on VSV-pseudotyped HIV-1 and wild-type HIV-1 infection in astrocytes, suggesting that infection is endocytosis-mediated. Also, entry restrictions that lead to minimal HIV-1 infection in primary astrocytes may not contribute to the overall viral load in the brain, although astrocytes serve as long-term viral reservoirs. We also found that astrocytes are infected only by high numbers of wild type HIV-1 particles (1.0 µg/mL), suggesting that HIV-1 infection in astrocytes is not a common phenomenon, at least in in-vitro studies on pure astrocytes and mixed brain cultures [8].

In an earlier study [73], we found that astrocytes could not be provoked by cytokines or proinflammatory molecules, which are elevated in the CSF of HIV-1-infected demented patients, to increase the efficiency of HIV-1 infection. However, the fact that lysosomotropic agents led to significant HIV-1 infection in astrocytes should serve as a caution against using such drugs on HIV-infected patients. These drugs could lead to unnatural infection in brain-resident astrocytes and other cells lacking HIV-1 receptors. Overall, the current findings elucidate the mechanism of Rev function in HIV-1 infected astrocytes. Current findings also demonstrate that natural infection by HIV-1 is minimally productive and that the functions of the regulatory proteins Tat and Rev were unperturbed.

## Materials and Methods

### Ethics Statement

Human fetal tissues at 10 to 12 weeks gestational age were obtained following written approval from adult female patients undergoing therapeutic abortion at the University of Washington, Seattle (IRB approval #11449) [8],[73]. The use of human fetal tissue was approved by the University of South Carolina (USCeRA#: HSA4636) and is IRB exempt (45 CFR 46.102(d) [8] [73]. All cell culture (primary human and cell lines), HIV infection, and HIV plasmid DNA studies were done according to University guidelines in a biocontainment facility approved by the Institutional Biosafety Committee (IBC) of the University of South Carolina.

### Primary human fetal brain and cell culture

Primary human fetal astrocytes (HFA) were cultured from human fetal tissues as described earlier [61],[73],[74],[75]. HFA, SVGA, SVGA-reporter cells (see **SI**), Jurkat, 293T (HEK), and HeLa cells were cultured. Human fetal brain specimens were obtained from the University of Washington, Seattle, where, following institutional guidelines, they were collected from fetuses at 8–12 weeks of gestational age. Briefly, the brain tissue was mechanically dissociated in Opti-MEM with 5% heat inactivated fetal bovine serum (FBS) and antibiotic solution (penicillin G, 100 units/ml; streptomycin, 100 µg/ml; and amphotericin B, 25 µg/ml). The dissociated cells (without trypsin) were cultured in Dulbecco’s modified Eagle’s medium (DMEM) with 10% FBS and antibiotics for at least one month before use. After 8 weeks in culture, HFA, after the 3^rd^ or 4^th^ passage, were used in experiments requiring a minimally non dividing population of astrocytes and no minor contaminants (neurons and microglia). In some experiments, HFA cultures were also treated with 5 mM LME (Sigma) for 8 h to remove any contaminating microglia [76]. The cultures were verified for purity using GFAP, MAP-2, and CD68 markers; no microglia were found, but a few neurons were always observed. To reduce the chance of mycoplasma contamination in these cultures, plasmocin, an antimycoplasma agent (Invitrogen, CA), was used during the initial culture passages. Cultures that tested negative for mycoplasma species on polymerase chain reaction were used.

SVGA (human astrocytic cell sub clone of SVG cells, a gift from Dr. Eugene Major, NIH) [74], SVGA- and HeLa-reporter cells (see **SI**) were maintained in DMEM supplemented with 2 mM L-glutamine, 10% FBS, and antibiotic solution. Jurkat and 293T cells were grown in RPMI-1640 supplemented with 10% FBS and 10% human serum as described earlier [73].

### Viral constructs and plasmids

HIV-1 Tat of 101 amino acids and mutant Tat (d-Tat) with 48–56 amino acids deleted, were cloned into pCDNA-3 vector [18],[59],[74], LTR-gag-GFPRRE, and Rev-BFP obtained from the QBIO lab. An HIV-1 LTR GFP was created by cloning HIV-1 LTR into CMV promoter-deleted pEGFP vector; Tat-GFP fusion vector was created in pEGFP vector [74]. LTR-RFP was created by cloning the RFP gene downstream of HIV-1 LTR in place of the removed CAT gene in a pHIV-LTR-CAT vector. Reporter construct CMV-gag-GFPRRE was created in a pcDNA-3 vector by insertion of a gag-GFPRRE cassette at BamH1 and EcoRV sites [18]. The green fluorescent reporter HIV-1 infectious molecular clone NLENY1 was created by inserting the YFP gene between the viral envelope and nef genes without impairing the viral open-reading frames, as reported earlier [60]. DDX-3 (wt), mutant DDX3, antisense DDX3 (AS-DDX3) [21] and TRBP constructs are in pcDNA. The NIH AIDS research and reagent facility provided pCV-1 (Rev and Tat co-expressing vector), and VSV-G expression vector, HIV-1 expression vector pNL4-3 (Malcolm Martin, NIH) [77], pMtat(-) HIV-1 molecular clone mutated for tat (R. Gallo, NIH), pMrev(-) from (Dr. Reza Sadaie) and infectious molecular clone YU-2 [78],[79]. Wild-type RNA helicase-A (RHA), mutant RHA were obtained from Toshihiro Nakajima (Japan) [58].

### Flow cytometry and reporter cells for HIV-1 Tat and Rev transcriptional regulation

HIV-1 LTR reporters were established in SVGA cells [74]. Stable LTR-GFP, LTR-RFP, or LTR-gag-GFPRRE with or without LTR-RFP plasmids in SVGA [8] or HeLa cells were attained by transfection. To create double Tat and Rev-responsive reporter cells, SVGA-LTRRFP cells were transfected with LTR-gag-GFPRRE constructs. The clonally selected single-cell populations were expanded, verified, and stored in liquid nitrogen. These astrocytic reporter cells were used to monitor HIV-1 LTR transcriptional activities of Tat and Rev either by HIV-1 infectious molecular clone transfection or HIV-1 infection. HIV-1 LTR transactivation was monitored by fluorescent microscopy, flow cytometry, or florimeter (Molecular Devices) at an excitation wavelength of 480 nm and emission of 530 nm. After trypsinization and washing, cells were fixed in 2% PFA. Reporter cells in PBS were then analyzed on FACS 440 (Becton Dickinson) [18],[59].

### Reverse genetics and VSV-G pseudotyping

HIV-1 NL4-3, NLENY1, and YU-2 were packaged in 293T cells with native HIV-1 envelope or pseudotyped with VSV-G envelope. Viral packaging was done with 10–17 µg of each lentiviral construct and 3–4 µg of VSV-G plasmid DNA, using Lipofectamine plus or Lipofecamine-2000 (Invitrogen) as described earlier [7],[18],[59]. After 16 h of transfection, cells in 100-mm dishes were washed twice with fresh medium and incubated in RPMI 1640 containing 10% FBS. Culture supernatants were harvested 72–80 h after transfection, centrifuged at 1,200 rpm for 15 min, and filtered through a 0.20 µm filter. The filtrate, after the addition of MgCl2 (4 mM), was digested with 10–50 units of RNase-free DNase (Invitrogen) per 1 µg of plasmid DNA for 30 min at 37°C, then aliquoted and stored at −80°C. The viral stocks were quantified for p24 antigen by ELISA (ZeptoMetrix). A viral inoculum of 50–1,000 ng/mL was used to infect lymphocytes, HFA, and SVGA.

### Virus infection

These studies used lymphocytes, HFA, SVGA, SVGA LTR-GFP, SVGA LTR-RFP, SVGA LTR-gag-GFP, or SVGA LTR-RFP/LTR gag-GFP reporter cells. NL-4-3, NLENY1, and VSV-pseudotyped HIV-1 viral stocks were prepared by transfection of 293T cells. An equal amount of virus from each strain (50–1000 ng/mL) was used to infect lymphocytes and astrocytes. About 800 µl of working viral stock was added to 60%–70% confluent 6-well culture plates and kept for 2 h at 37°C with gentle mixing every 20 min. After 2 h of incubation, the inoculum was washed two times with DMEM and complete medium was added. The next day, cultures were washed two times and replenished with complete medium, then followed up for 3–16 weeks by monitoring green fluorescence, cytopathic effect (CPE), and p24. 50,000–75,000 HFA were seeded in 6-well tissue culture plates. On the third day, duplicate samples were pretreated for 2 h with Dynasore (80 µM) and 0.2% DMSO. This was followed by HIV-1 infection for 2 h. HFA were washed two times with culture medium to remove residual viral activity. We measured the dose response for toxicity of the inhibitor by Quant-Blue cell viability assay (Bioassay System Hayward, CA) and LDH assay [7] [59] [73]. The gold standard for toxicity was the Trypan blue dye exclusion test. Supernatants were collected for determination of p24 levels by ELISA.

### Transfection of plasmids and siRNA

HFA, SVGA, SVGA reporter cells, and HeLa cells were transfected with HIV-1 infectious molecular clones, Rev, Tat, dTat, DDX3, TRBP expression vectors, or reporter plasmids, using Lipofectamine 2000 (Invitrogen). The transfection efficiency was ∼80%, but was remarkably low in primary astrocytes. The plasmid concentrations used ranged from 0.5 to 15 µg, depending on the culture plates used. siRNAs were designed by web-based siRNA programs (Ambion and Dharmacon); sequences were synthesized (Dharmacon) or purchased (Ambion) (see **SI**). Transfection of siRNAs was done together with plasmids whenever necessary. We used lipofectamine-2000 for siRNA transfection and rhodamine (red fluorescent) labeled nonspecific siRNA to determine transfection efficiency (Dharmacon and Invitrogen). PKR, and scrambled siRNA sequences were purchased from Dharmacon (see **SI**).

### HIV-1 and cellular protein analysis by immunofluorescence and Western blotting

Tat and p24 expression in HIV-1 infected or transfected astrocytes, Hela, and lymphocytes was detected by immunofluorescence using HIV-1 p24 Gag monoclonal antibody, and Tat antibodies (ABI). Infected lymphocytes and transfected cells were fixed in 2% paraformaldehyde in PBS (pH 7.2) for 15 min, then permeabilized with 0.2% Triton-X-100 (PBS) for 11 min. This was followed by blocking with 5% BSA in PBS for 1 h at 25°C. The treated cells were overlaid with primary antibodies at optimum working dilutions of Tat 1∶400, and p24 1∶200 in a moist chamber at 4°C overnight. After washing four times with PBS (10 min each at 37°C), the secondary antibodies (Molecular probes) labeled with Alexa 568 or 488 (1∶500 dilutions) were added to the specimens for 35 min at 25°C in the dark. Subsequently, the specimens were washed four times with PBS, stained with DAPI (PBS) for 10 min during the last wash, rinsed with PBS, and mounted with anti-fading agent (biomeda). The stained specimens were stored at 4°C or −20°C (long-term) until examined. The specimens were visualized using a UV microscope (Nikon). Images were captured with a digital camera (Nikon).

Western blotting for various gene products, either constitutive or after ablation by siRNAs, was done on astrocytes, Hela cells, Jurkat, and 293T. Each sample of 30–40 µg total protein was analyzed on SDS-PAGE and electroblotted to a PVDF membrane. The immunoblotting antibodies for Tat (ABI); RHA and DDX-1 (abcam); PKR and DDX-3 (Cell signaling); Hax-1 (BD Biosciences); Sam-68 (Upstate Cell Signaling); TRBP (IMGENEX), and actin (Sigma) were used. We used these antibodies in combination with HRP-labeled secondary antibodies (BioRad). The blots were exposed to X-ray films and quantified by NIH software.

### Virus release assay (p24)

Astrocytes and HeLa cells seeded at 60%–70% confluency in culture dishes or on plates were transiently transfected with 2.5–7.5 µg of HIV-1 infectious molecular clones (YU-2, NL4-3, NLENY1, pMrev(-) or pMtat(-)), using Lipofectamine 2000 (Invitrogen) as described [61] [74]. At various time points, the culture fluids were monitored for p24. Quantitative viral p24 titration was done by ELISA (ZeptoMetrix). Data were expressed as mean ± SEM. Multiple infection and transfection experiments (n≥5) were done.

### Statistics

Results are represented as mean ± standard error of the mean (SEM) for each bar set plotted using Sigma plot v12.0 with associated p-values for each treatment group compared to its control. Statistical analysis was done using Origin 6.1 software. Significance between two groups was calculated using the two-tailed Student’s t-test p<0.05 was considered significant.

## Supporting Information

Supporting Information S1 **HIV-1 inhibitors with working concentrations, reporter cell lines, and custom made siRNA sequences and PCR primer sequences are provided.**
(DOCX)
