# Supplementary material for: Unperturbed Posttranscriptional Regulatory Rev Protein Function and HIV-1 Replication in Astrocytes
Source: PLoS One. 2014 Sep 4;9(9):e106910. doi: 10.1371/journal.pone.0106910 (PMC4154834; doi:10.1371/journal.pone.0106910)
Supplement: Supporting Information S1 — HIV-1 inhibitors with working concentrations, reporter cell lines, and custom made siRNA sequences and PCR primer sequences are provided. (DOCX) [file pone.0106910.s001.docx]

**Table 1**

HIV-1 activators and inhibitors

| Activators/Inhibitors | Action | HIV status (Conc.) |
| --- | --- | --- |
| Chloroquine | Increase in endosomal pH | Upregulation (10 µM) |
| Bafilomycin-A | Increase in endosomal pH | Upregulation (100 nM) |
| Leptomycin B | Inhibition of Rev function | Inhibition (10 nM) |
| Dynasore | Dynamin inhibitor | Inhibition (80 µM) |
| TNPO3 | Viral DNA import inhibition | Inhibition (50 nM) |
| PKR-siRNA | Antiviral | Upregulation (100 nM) |
| Control siRNA | Negative control | No effect (100 nM) |

**Table 2**

Tat and Rev-responsive HIV-1 LTR reporter cells

| Reporter plasmid (s) | Cells type | Purpose |
| --- | --- | --- |
| LTR-EGFP | SVGA | Tat biological activity |
| LTR-RFP | SVGA | Tat biological activity |
| LTR-gagGFPRRE | SVGA | Rev biological activity |
| LTR-gagGFPRRE | HeLa | Rev biological activity |
| LTR-gagGFPRRE/LTR-RFP | SVGA | Tat/Rev biological activity |

**Table 3**

Custom design siRNA sequences used

| **Target gene** | **siRNA sequence** |
| --- | --- |
| TNPO3 | sense 5’-CGACAUUGCAGCUCGUGUAUU-3’  antisense 5’-UACACGAGCUGCAAUGUCGUU-3’ |
| DDX1 | , sense 5’-CGGUGUUCCUUAUGUUAUAdTdT-3’  antisense 5’-UAUAA; CAUAAGGAACACCGdTdT-3’ |
| TRBP | , sense 5’-GCAAUGAGGUGGAGCCUGAUU-3’  antisense 5’-GCAAGAAGGCAGCCAAGCAUU-3’ |
| PKR | Dharmacon (cat # P-002028-01) |
| Control siRNA | Dharmacon (cat. #D-001600-01-05) |

**Table 4**

Primers used in viral DNA integration

| **Genes** | **Primer sequences** |
| --- | --- |
| GAPDH | GAPDH-f5’-CATCAGCAATGCCTCCTGCACC-3’  GAPDH-r 5’-GTGCTCAGTGTAGCCCAGGATG-3’ |
| Flanking primers | INT-1, 5’-TGCTGGGATTACAGGGCGTGAG-3’  INT-2, 5’-TAGACCAGATCTGAGCCTGGGA-3’ |
| Nested primers | INT-N1, 5’-GGCTAACTAGGGAACCCACTG-3’  INT-N9, 5’-CTGCTAGAGATTTTCCACACTGAC-3’ |
